# Real‐time 4D dose reconstruction for tracked dynamic MLC deliveries for lung SBRT

**DOI:** 10.1118/1.4965045

**Published:** 2016-10-21

**Authors:** Cornelis Ph. Kamerling, Martin F. Fast, Peter Ziegenhein, Martin J. Menten, Simeon Nill, Uwe Oelfke

**Affiliations:** ^1^ Joint Department of Physics, The Institute of Cancer Research and The Royal Marsden NHS Foundation Trust, London SM2 5NG, United Kingdom

**Keywords:** cancer, collimators, computerised tomography, dosimetry, image reconstruction, medical image processing, Monte Carlo methods, radiation therapy, tumours, Dose‐volume analysis, Monte Carlo methods, Collimators, Reconstruction, Computed tomography, Cancer, Computerised tomographs, Radiation therapy, Digital computing or data processing equipment or methods, specially adapted for specific applications, Image data processing or generation, in general, Using diaphragms, collimators, Scintigraphy, real‐time dose reconstruction, 4D dose calculation, MLC tracking, lung SBRT, Dosimetry, Multileaf collimators, Lungs, Cancer, Real time information delivery, Medical imaging, Computer software, Tissues, Linear accelerators

## Abstract

**Purpose:**

This study provides a proof of concept for real‐time 4D dose reconstruction for lung stereotactic body radiation therapy (SBRT) with multileaf collimator (MLC) tracking and assesses the impact of tumor tracking on the size of target margins.

**Methods:**

The authors have implemented real‐time 4D dose reconstruction by connecting their tracking and delivery software to an Agility MLC at an Elekta Synergy linac and to their in‐house treatment planning software (TPS). Actual MLC apertures and (simulated) target positions are reported to the TPS every 40 ms. The dose is calculated in real‐time from 4DCT data directly after each reported aperture by utilization of precalculated dose‐influence data based on a Monte Carlo algorithm. The dose is accumulated onto the peak‐exhale (reference) phase using energy‐mass transfer mapping. To investigate the impact of a potentially reducible safety margin, the authors have created and delivered treatment plans designed for a conventional internal target volume (ITV) + 5 mm, a midventilation approach, and three tracking scenarios for four lung SBRT patients. For the tracking plans, a moving target volume (MTV) was established by delineating the gross target volume (GTV) on every 4DCT phase. These were rigidly aligned to the reference phase, resulting in a unified maximum GTV to which a 1, 3, or 5 mm isotropic margin was added. All scenarios were planned for 9‐beam step‐and‐shoot IMRT to meet the criteria of RTOG 1021 (3 × 18 Gy). The GTV 3D center‐of‐volume shift varied from 6 to 14 mm.

**Results:**

Real‐time dose reconstruction at 25 Hz could be realized on a single workstation due to the highly efficient implementation of dose calculation and dose accumulation. Decreased PTV margins resulted in inadequate target coverage during untracked deliveries for patients with substantial tumor motion. MLC tracking could ensure the GTV target dose for these patients. Organ‐at‐risk (OAR) doses were consistently reduced by decreased PTV margins. The tracked MTV + 1 mm deliveries resulted in the following OAR dose reductions: lung *V*_20_ up to 3.5%, spinal cord *D*_2_ up to 0.9 Gy/Fx, and proximal airways *D*_2_ up to 1.4 Gy/Fx.

**Conclusions:**

The authors could show that for patient data at clinical resolution and realistic motion conditions, the delivered dose could be reconstructed in 4D for the whole lung volume in real‐time. The dose distributions show that reduced margins yield lower doses to healthy tissue, whilst target dose can be maintained using dynamic MLC tracking.

## 1. INTRODUCTION

Randomized controlled trials have shown that dose escalation in stereotactic body radiation therapy (SBRT) for stage I nonsmall cell lung cancer results in high tumor control (>90% for primal local tumor control).^1^ A stage I lung tumor typically moves a few millimeters up to a few centimeters due to respiratory motion.^2^ Assuring dose coverage of the tumor can be accomplished by deep‐inspiration breath‐hold or respiratory gating strategies,^3^ at the cost of patient discomfort, longer treatment times, and requiring the patient's collaboration. The motion can also be incorporated into treatment planning, by constructing a planning target volume (PTV) based on an internal target volume (ITV). The ITV is defined as the composite volume of gross target volumes (GTVs), delineated on various phases of a 4DCT reflecting the breathing cycle.^4^ Although this straight‐forward approach guarantees target coverage for the whole breathing cycle (as long as the motion in treatment and imaging sessions coincide), the high‐dose volume is unnecessarily large and potentially toxic to surrounding normal tissues. A refinement of this approach is the midventilation (midV)/midposition (midP) approach^5^, ^6^ which is based on the average tumor position resulting in smaller target margins. Especially for hypofractionated treatment regimens, moving away from the very conservative ITV‐based PTV is expected to reduce toxicity.

Tracking was proposed to manage intrafractional motion as an alternative to breath‐hold and gating techniques.^7^ It does not affect patient comfort and has a minimal impact on treatment delivery time. Tumor tracking solutions for lung SBRT have been presented and evaluated for the robotic Cyberknife (Accuray Inc., Sunnyvale, CA, USA)^8^, ^9^ and the gimbaled Vero (Brainlab AG, Feldkirchen, Germany) machines.^10^, ^11^ Both machines are intentionally designed for tumor tracking by either allowing for moving the whole beam using a robotic arm or panning and tilting the beam utilizing gimbals. Alternatively, multileaf collimator (MLC) tracking was proposed as a tracking solution for conventional linacs.^7^, ^12^ The currently established method is centroid tracking, which aims at dynamically reshaping the treatment field in the beam's‐eye‐view according to the actual recorded target motion. The method was recently introduced clinically for prostate^13^ and lung SBRT.^14^ If deformations of the tumor are small, centroid tracking is expected to compensate for a large part of the motion. In this work we use an in‐house implementation of MLC centroid tracking^12^ for lung SBRT. We present an alternative to the ITV and midV planning approaches, which decreases the PTV size under the assumption MLC tracking corrects for rigid target volume shifts.

To assess the impact of MLC tracking on target coverage and organ‐at‐risk (OAR) dose, the expected deviation between the planned and delivered dose must be quantified. Several methods have been proposed to compute the delivered dose based on offline dose reconstruction.^15^, ^16^, ^17^, ^18^ Recently, we have presented an online dose reconstruction solution for assessment of prostate SBRT,^19^ which calculates and accumulates dose in real‐time based on dose‐influence data, while accounting for the machine/target motion interplay. Dose‐influence data describe the influence of the fluence distribution on the patient dose. To make this approach applicable to dose calculation, the fluence distribution is subdivided into small rectangular segments, called bixels. Online dose reconstruction provides real‐time quality assurance during treatment and provides the means to directly validate the performance of MLC tracking. Moreover, it is a prerequisite for replanning scenarios in which the treatment plan is changed on the fly during delivery. For example, drift motion might result in a gradually changing trade‐off between target coverage and OAR dose which can be compensated for during delivery by replanning between beams.

Schmidt *et al.* ^20^ have performed a dosimetric analysis for lung radiation therapy (33 fractions) utilizing 4D offline dose reconstruction and use isocenter shifts to model the motion.^17^ We believe that although this method may be of value for treatment sites with homogeneous tissue and rigid motion (e.g., prostate), it is flawed by inaccuracies for large tissue inhomogeneities and deformations as found in the lung. The group found small changes to target coverage when motion was incorporated in the dose calculation model and concluded the effect of interfractional motion is larger than intrafractional motion. For hypofractionated treatments such as lung SBRT, however, intrafractional motion does not simply blur the delivered dose anymore and hence its accurate mitigation is of importance. The isocenter shift method from Poulsen *et al.* ^17^ is not suitable for estimation of OAR dose when target motion is different from OAR motion (as is the case in the lung). Moreover, this method is based on 3D image data, although 4D image data are usually available. Glitzner *et al.* ^21^ have proposed a 4D dose reconstruction pipeline for kidney based on online MR imaging and generation of a pseudo 4DCT. They use deformable vector fields (DVFs) to map dose from each phase to a reference phase. Their dose calculation algorithm takes 15 s/MLC aperture and hence cannot be utilized in an online dose reconstruction scenario.

To facilitate a 4D online dose reconstruction solution for lung SBRT, we identified the following challenges. To incorporate the respiratory motion, dose calculation has to be based on 4D images. Moreover, dose has to be calculated for each MLC aperture using a Monte Carlo method due to considerable tissue inhomogeneities often encountered in lung. Furthermore, accumulating the dose onto a reference phase requires deformable image registration (DIR) and a real‐time dose mapping algorithm. We add to our previously presented online dose reconstruction platform^19^ by providing implementations for each of these challenges.

The goal of this work is two‐fold. Firstly, we present a fully implemented method to perform real‐time 4D dose reconstruction and show the runtimes of dose calculation and accumulation. Secondly, we assess whether safety margins can potentially be reduced when dynamic MLC tracking is performed for lung SBRT. We have created treatment plans with various margins for four patients with stage I tumors and different motion conditions captured on a 4DCT. We have delivered the plans with and without MLC tracking on a linac and have assessed the online reconstructed dose.

## 2. MATERIALS AND METHODS

### 2.A. Real‐time dose reconstruction platform

**Figure 1:**
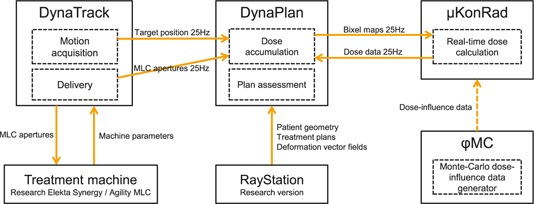
This component diagram shows how the software modules are interfaced to facilitate online 4D dose reconstruction. The solid arrows represent direct interfaces, the dashed arrow represents an indirect (i.e., file‐based) interface.

The 4D online dose reconstruction components presented in this manuscript were built on top of the software platform we have presented in Ref. 19. Figure 1 shows the different components and data transfers as used for this work.

#### 2.A.1. Motion acquisition and delivery

The tracking and delivery software DynaTrack^12^ is connected to the research version of an Elekta AB (Stockholm, Sweden) Synergy linac with Agility MLC through a proprietary real‐time network interface. DynaTrack is able to receive motion acquisition data from a variety of peripheral systems. Motion acquisition was based on simulated data for this work.

#### 2.A.2. Dose calculation/accumulation/assessment

DynaTrack is connected to the treatment planning software (TPS) DynaPlan^22^ through a TCP/IP network interface. DynaPlan receives actual target positions and MLC apertures independently at 25 Hz, and is responsible for dose accumulation. To calculate the dose for a given MLC aperture, DynaPlan utilizes the real‐time *μ*KonRad^23^ dose calculation module, which exploits precalculated dose‐influence data. These data were generated in *ϕ*MC, a fast central processing unit (CPU)‐based Monte Carlo dose calculation engine.^24^

#### 2.A.3. RayStation c++ interface

DynaPlan directly interfaces RayStation 4.6 (research version, RaySearch Laboratories, Stockholm, Sweden) through an in‐house native c++ application program interface,^25^ which allows importing patient geometries, treatment plans, and DVFs. The patient geometry and DVFs are utilized to accommodate real‐time dose accumulation in DynaPlan and *μ*KonRad. The treatment plans are processed in DynaTrack.

### 2.B. Real‐time energy‐mass transfer

Conventional TPSs utilize direct dose mapping (DDM) to perform dose accumulation for deformed geometries. A shortcoming of DDM is that it is based on interpolation of dose values between grids. Dose mapping by energy‐mass transfer (EMT)^26^ maps energy and mass contribution separately and is therefore based on the physical definition of dose. Li *et al.* ^27^ have analyzed the dosimetric differences between DDM and EMT for several lung cases and conclude that although mean PTV dose values are similar, significant dose deviations can occur for local dose features. Therefore, DDM is deemed inaccurate for applications in which minimum PTV dose plays an important role.

For this work we have adapted the EMT pseudocode provided by Li *et al.* ^27^ to facilitate real‐time dose accumulation. Although the operational semantics of the algorithm were not changed, it was highly optimized to benefit from CPU caching, thread‐level (using OpenMP)^28^ and data‐level (using AVX) parallelization. In line with the analysis of Li *et al.*,^27^ a dose grid resolution of ∼2 × 2 × 2 mm^3^ voxels and CT grid / DVF resolution with ∼1 × 1 × 2 mm^3^ voxels was used.

### 2.C. Simulation of motion trajectory

**Figure 2:**
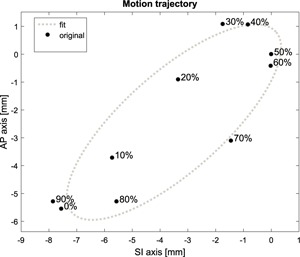
Motion trajectory for patient 3, based on the center of volume of individual GTVs.

For each patient, a tumor trajectory was generated based on the respective phase‐binned 4DCT. An example trajectory is shown in Fig. 2. An ellipse was fitted to the superior–inferior (SI) and anterior–posterior (AP) center‐of‐volume positions of all GTVs using a least squares estimator. Based on the ellipse fit and assuming a respiratory period of 5 s, a 2D sinusoid motion trajectory was created using 25 Hz sampling. Left–right (LR) motion was ignored as it was smaller than 3 mm in all cases. Each trajectory was then used as input for the motion acquisition module of DynaTrack during delivery.

DynaTrack was extended to compute the respiratory phase of each acquired target position in real‐time. The phase‐binning algorithm is based on previous work by Lu *et al.* ^29^ but was modified to allow for online binning. Bins are defined relative to the automatically detected extremal respiratory positions (“peaks” and “valleys”) in each respiratory cycle. During real‐time motion acquisition and binning, future data points are not available after acquiring the current data point making it impossible to use the current respiratory branch for binning. Instead the data point is phase shifted by one respiratory cycle, and binned relative to the last fully acquired exhalation or inhalation branch. The respiratory period is calculated from the last 20 s worth of motion data using Fourier analysis.

### 2.D. Data dependencies for online calculations

**Figure 3:**
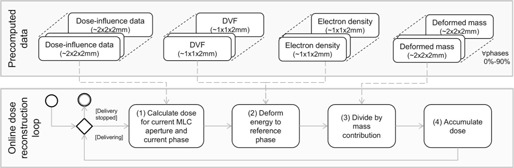
The bottom box contains an activity diagram representing the steps of the online 4D dose reconstruction loop. The loop is performed for each received MLC aperture. The individual steps are described in more detail in Sec. 2.D. The top box shows all data which are precomputed for each phase before online dose reconstruction is performed. The dashed arrows show the relation between the precomputed data and the dose reconstruction steps.

Figure 3 presents the dependencies on precalculated data in the steps of the dose reconstruction algorithm. The activity diagram in the online dose reconstruction loop box shows how dose is accumulated during delivery. The dashed arrows relate the precomputed data to the online step. For each received MLC aperture, (1) its dose contribution has to be computed by converting the aperture to beamlet weights and subsequently multiplying with the dose‐influence data for the respective beam and phase. (2) The energy from the dose contribution is then computed using the precomputed high‐resolution DVFs and the electron density of the respective phase. (3) The energy then needs to be divided by the deformed mass of the respective phase. (4) Finally, the dose can be accumulated to the previously delivered dose.

### 2.E. Moving target volume

**Figure 4:**
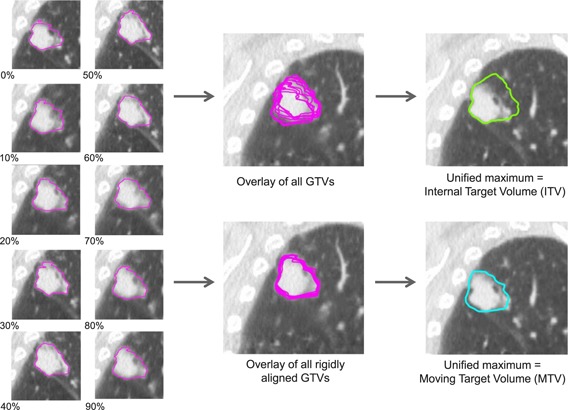
The ITV (green contour) is constructed as the unified maximum of the GTVs (magenta contours) of all ten 4DCT phases. The MTV (cyan contour) is constructed as the unified maximum of rigidly aligned GTVs, based on their center of volume. The CT images in the center column show the reference phase (peak‐exhale). The MTV is shown for a single phase only and is shifted for all other phases. The ITV is constant for all phases. The 4DCT data correspond to patient 3.

Assuming that centroid MLC tracking compensates for rigid target shifts, we have created treatment plans for a tracking scenario using the moving target volume (MTV) approach, which was utilized in Menten *et al.* ^30^ Figure 4 schematically shows how construction of the MTV can be compared to the construction of the conventional ITV. Both methods rely on the GTV being delineated on each individual 4DCT phase. An ITV is then constructed by taking the unified maximum of all GTVs. For the MTV method, all GTVs are first rigidly aligned to the reference GTV, based on the center of volume. The MTV is then constructed by taking the unified maximum of the shifted GTVs.

### 2.F. Patient cohort and motion conditions

**Figure 5:**
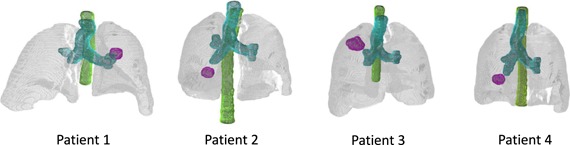
Volume renderings of the patient geometries, showing: lungs (gray), proximal airways (cyan), spinal cord (green), and GTVref (magenta) for the reference phase.

Table I describes the patient data for the cohort analyzed in this work. Four patients with different tumor motion properties were selected, which were previously treated with lung SBRT in our clinic. All tumors were located peripherally as defined by RTOG 1021. Figure 5 shows the location of the GTV in the patient for the reference phase. The GTVs were manually delineated for each individual 4DCT phase. The GTV maximum motion per direction (SI, AP, and LR) was calculated in RayStation based on the center‐of‐volume shifts. The minimum (min), maximum (max), and reference (ref) GTV volumes give indication for the tumor deformation. The resolution was based on the 4D planning CT with 512 × 512 voxels/slice. Dose computation and accumulation were performed on a downsized grid with 256 × 256 voxels/slice using dose‐influence data ranging from 18 to 47 GB/patient. Dose accumulation was performed for the whole patient volume between the first and last slice containing lung tissue. Each 4DCT phase was registered to the reference phase (peak exhale) using RayStation's hybrid DIR module,^31^ which utilizes image intensities, lung delineations, and patient contours. The accuracy of DIR was established by visual assessment.

**Table I:** Patient cohort descriptors.

		Peak‐to‐peak motion (mm)				GTV volume (cm^3^)					
Patient	Tumor location	SI	AP	LR	3D	Min	Max	Ref	Lung slices	*D_ij_* size (GB)	Resolution CT grid (mm^3^)
1	Left upper lobe	5.5	2.2	1.2	6.0	9.7	14.5	9.7	116	18	1.37 × 1.37 × 2.00
2	Right lower lobe	13.9	2.6	0.4	14.1	2.7	3.6	3.3	122	21	1.05 × 1.05 × 2.00
3	Right upper lobe	7.8	6.6	2.6	10.5	23.6	28.5	25.6	118	47	0.98 × 0.98 × 2.00
4	Right lower lobe	5.5	3.0	0.6	6.3	8.4	9.9	8.6	128	40	0.98 × 0.98 × 2.00

### 2.G. Treatment planning rationale

For each patient, treatment plans were created using RayStation for five different PTVs. First, the conventional ITV approach with an isotropic 5 mm margin PTV_ITV +5_ was applied for reference. Then, MTVs were created for each patient by RayStation scripting, and subsequently 5, 3 and 1 mm isotropic margins were applied resulting in three additional PTVs: PTV_MTV +5_, PTV_MTV +3_, and PTV_MTV +1_. All ITV and MTV treatment plans were designed on the reference phase. Finally, a midV plan was generated on the midV phase as described by Wolthaus *et al.* ^5^ The PTV_midV_ for these plans was generated by expanding the GTV by 4.4–7.6 mm depending on the intrafractional motion observed in the 4DCT scan. All plans were designed for step‐and‐shoot IMRT with nine equidistant beams according to RTOG 1021 (3‐Fx) with a *D*_95_ prescription of 18 Gy/Fx. Delivery was performed using 6 MV beams with flattening filter at a 550 monitor units (MU)/min dose rate.

The ITV+5 and midV plan were delivered in two modes:

**Static ITV+5/midV:** Actual target position data are ignored for both delivery and dose accumulation. For ITV+5, the dose is calculated based on the dose‐influence data for the reference phase only. For midV, the dose is calculated for the midV phase, respectively. The accumulated dose represents the planned dose distribution for PTV_ITV +5_/PTV_midV_.

**Conventional ITV+5/midV:** Actual target position data are ignored for delivery, but incorporated in dose accumulation: the dose is calculated based on the dose‐influence data for the reported phase and warped onto the reference phase using EMT. This mode simulates current clinical practice: substantial motion occurs, but ITV/midV margins prevent cold‐spots in the dose to the GTV.

To assess target coverage and potential dose reduction to OARs for the MTV plans compared to the ITV+5/midV plans, all MTV plans were delivered in tracked mode:

**Tracked MTV:** Actual target position data are used for centroid MLC tracking and incorporated in dose accumulation. The dose is calculated based on the dose‐influence data for the reported phase and warped onto the reference phase using EMT.

To assess the fidelity of tracking and dose mapping from a technical point of view, we have also analyzed the MTV plans in nontracked mode:

**Static MTV:** Technically the same as static ITV+5/midV. This represents the planned dose distribution for PTVMTV+1,3,5.

**Untracked MTV:** Technically the same as conventional ITV+5/midV. This mode simulates what happens when margins are decreased without tracking: it is expected that substantial motion degrades the planned dose distribution.

We have compared the untracked and tracked MTV dose to the static MTV dose. It is expected that when untracked delivery leads to inadequate GTV coverage, this can be corrected by tracking.

### 2.H. Delivery analysis

To assess the dosimetric performance of MLC tracking, four dose‐volume histogram (DVH) points were selected for analysis and computed for the reconstructed dose on the reference phase. To assess target coverage, *D*_98_ GTV_ref_ was computed (*D_x_* being defined as the dose exposed to *x*% of a volume). To assess dose spillage to the lung, *V*_20_ was evaluated.^32^ *V*_20_ was computed for the lung volume, after subtracting GTV_ref_ (*V_x_* being defined as the volume exposed to a dose ≥*x* Gy of a volume‐of‐interest). Moreover, *D*_2_ was evaluated for spinal cord and the proximal airways.

Decreasing PTV margins for a constant PTV dose description may result in a decreasing GTV dose. To provide a fair analysis of OAR dose reduction, we have normalized the dose distribution: *D*_98_ for the GTV for all MTV plans was scaled to GTV *D*_98_ for the conventionally delivered ITV+5 plan.

To show whether dose calculation and accumulation can be performed before the next MLC aperture arrives (i.e., within 40 ms), we have measured the respective runtimes. For each patient, mean, 5%‐, and 95%‐percentiles (5%‐per, 95%‐per) were computed for dose calculation and accumulation separately for the MTV+1 plan using tracked delivery. The total runtime includes dose calculation, dose accumulation, MLC aperture to beamlet conversion, and logging.

### 2.I. Hardware/software configuration

For this work, DynaPlan was running on an Intel (Santa Clara, CA, USA) Xeon E5‐2697 v3 2.6 GHz in dual configuration with 128 GB main memory. DynaTrack was running on an Intel Xeon E5‐2620 2.0 GHz. Both were compiled using Microsoft (Redmond, WA, USA) Visual Studio 2010/2012 and ran on the Microsoft Windows 7 operating system. All high‐performance algorithms were developed to run on Intel Xeon multicore CPU systems.

## 3. RESULTS

### 3.A. Runtime analysis

Table II summarizes the runtimes for the full real‐time computation required for each incoming MLC aperture in DynaPlan. The mean runtime for dose calculation and accumulation ranged from 7.3 to 14.9 ms and 13.1 to 9.1 ms, respectively. The mean total runtime ranged from 21.3 to 34.5 ms. The 95%‐percentile ranged from 25.5 to 41.1 ms.

**Table II:** Runtime analysis.

	Patient 1			Patient 2			Patient 3			Patient 4		
	5%‐per	Mean	95%‐per	5%‐per	Mean	95%‐per	5%‐per	Mean	95%‐per	5%‐per	Mean	95%‐per
Dose calculation (ms)	6.8	7.9	11.2	5.3	7.3	11.3	12.5	14.9	18.7	9.5	12.0	15.1
Dose accumulation (ms)	11.1	13.1	15.6	12.5	15.4	18.8	15.8	19.1	23.1	12.2	14.9	18.9
Total (ms)	18.5	21.3	25.5	19.0	23.1	31.8	29.7	34.5	41.1	23.2	27.2	32.9

### 3.B. Dosimetric impact of intrafractional motion and MLC tracking

**Figure 6:**
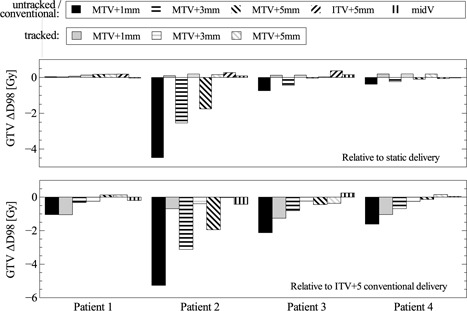
Dosimetric impact of intrafractional motion and MLC tracking for different plans and delivery modes. The first row indicates target coverage relative to the respective static (planned) case. The second row shows target coverage relative to the conventional ITV+5 case.

The dosimetric results for GTV *D*_98_ are summarized in Fig. 6. All bars with a black texture represent conventional deliveries, all gray textures the tracked deliveries. All dose differences are based on dose per fraction and are computed on the reference phase.

The first row shows the dose difference for GTV *D*_98_ relative to the static (planned) target dose. For patient 1, all dose differences were negligible (<0.19 Gy). For patient 2–4 and all MTV plans, *D*_98_ decreased up to 4.5 Gy for the untracked delivery, but could be restored to an increase of 0.07–0.19 Gy by MLC tracking. The deviations for the midV plans were small for all patients: −0.02 to 0.15 Gy.

The second row shows the dose difference from GTV *D*_98_ relative to the conventionally delivered ITV+5 plan. For the MTV+3 and MTV+1 plans tracked delivery resulted in a *D*_98_ decrease of 0.24–1.3 Gy. For the MTV+5 plans, Δ*D*_98_ ranged from −0.37 to 0.16 Gy. The deviations between the conventionally delivered midV and ITV+5 plans were −0.42 and 0.25 Gy.

**Figure 7:**
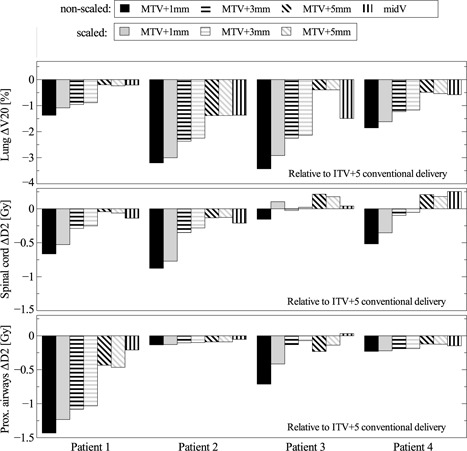
Dosimetric impact of intrafractional motion and MLC tracking for different plans, both nonscaled and scaled to GTV *D*_98_ for the conventionally delivered ITV+5 plan. The dose differences are compared to the conventional ITV+5 case for lung *V*_20_, spinal cord *D*_2_, and proximal airways *D*_2_.

The dosimetric results on OARs for the tracked deliveries are summarized in Fig. 7. All dose differences are based on dose per fraction and are computed on the reference phase. To obtain *V*_20_, the fraction dose was normalized to total dose. All bars with a black texture refer to nonscaled dose distributions, all gray textures represent the dose distributions after scaling GTV *D*_98_ to GTV *D*_98_ from ITV+5 conventional.

The first row shows the relative differences for lung *V*_20_ compared to the ITV+5 plan, conventionally delivered. For all patients and plans *V*_20_ decreased. For tracked MTV+1, MTV+3, and MTV+5, the decrease ranged from 1.4%–3.5%, 1.0%–2.4%, and 0.2%–1.5% respectively. For the conventional midV plans, the decrease was 0.2%–0.6% for patient 1 and 4, and 1.4%–1.5% for patient 2 and 3.

The second row presents the relative differences for spinal cord *D*_2_. For all tracked MTV+1 and MTV+3 plans, *D*_2_ decreased compared to the conventionally delivered ITV+5 plan. For tracked MTV+1 the decrease ranged from 0.15 to 0.90 Gy, for tracked MTV+3 from 0.021 to 0.35 Gy. For tracked MTV+5, Δ*D*_2_ for spinal cord ranged from −0.13 to 0.18 Gy. The deviations for the midV plans were −0.14 to 0.25 Gy.

The third row shows the relative differences for proximal airways *D*_2_ compared to the conventionally delivered ITV+5 plan. Similar to the spinal cord *D*_2_, for all tracked MTV+1 and MTV+3 plans, *D*_2_ decreased. For tracked MTV+1 the decrease ranged from 0.14 to 1.4 Gy, for tracked MTV+3 from 0.026 to 1.1 Gy. For tracked MTV+5, the decrease in *D*_2_ ranged from 0.029 to 0.44 Gy. The deviations for the midV plans were −0.21 to 0.03 Gy.

## 4. DISCUSSION

### 4.A. Technical analysis

We have successfully implemented online 4D dose reconstruction in our research treatment planning and delivery platform. The tracking solution can be used on a conventional linac. The tracking and delivery software DynaTrack runs on a computer in the linac control room, while the workstation running DynaPlan can be placed anywhere, as long as a low‐latency network connection is available. The algorithms for dose calculation and accumulation reach a high memory bandwidth of 50–70 GB/s on the workstation used in this study. Although the runtimes are reasonably stable, Windows is not a real‐time operating system. Hence there might always be an unexpected lag, resulting in individual outlier runtimes for a few scattered instances. The mean computation time per MLC aperture ranged from 21.3 to 34.5 ms which was well below 40 ms. Hence, a computation rate of 25 Hz can be maintained. If latency builds up nonetheless, it can be compensated during beam‐off periods, when no accumulation has to be performed. For the patient data in this study, the runtimes scale with the size of the dose‐influence data. On computers with less memory bandwidth or computational power, the number of slices to calculate and accumulate dose for could be decreased (e.g., to the target region‐of‐interest only, instead of the whole lung volume).

For this work, target position data were simulated in DynaTrack. However, the tracking delivery software supports various motion acquisition methods, like implanted electromagnetic transponders^33^ and ultrasound transducers.^34^ DynaTrack effectively compensates for the additional latency by prediction.^35^ The tumor trajectories in this study were generated based on 4DCT data by fitting an ellipse. The root mean square error of the fit error was 0.13–0.51 mm, which is deemed acceptable as it is substantially smaller than the voxel size utilized for dose reconstruction.

### 4.B. Dosimetric analysis

We could show that for all cases in which substantial motion led to inadequate target coverage for the untracked MTV plans, tracking could successfully recover the GTV cold‐spots. This effect was largest for patient 2 and 3, with peak‐to‐peak motion clearly exceeding the photon penumbra for lung tissue (assumed to be approximately 6.4 mm).^36^ GTV cold‐spots were not observed for patient 1, for which the GTV deformation was largest (49% increase compared to volume on reference phase). Due to the nature of the MTV approach, the PTV will be strongly influenced by the largest GTV and is therefore robust against motion. The GTV motion for patient 2–4 was more rigid and hence could be compensated for by MLC centroid tracking. This serves as a proof of concept for lung SBRT MLC tracking.

The assessment of lung *V*_20_, spinal cord *D*_2_, and proximal airways *D*_2_ shows that decreasing the PTV size decreases OAR dose, potentially reducing toxicity.^32^ The dose reductions for spinal cord and proximal airways should be related to the RTOG 1021 dose constraints. For the spinal cord maximum point dose is defined as 7.3 Gy/Fx and the volume exceeding 4.1 Gy/Fx should be <1.2 cm^3^. The evaluated spinal cord *D*_2_ ranged from 3.1 to 3.9 Gy for the ITV+5 conventional delivery. For some tracked cases *D*_2_ for spinal cord was slightly higher than for the conventional ITV+5 delivery. These differences are however ≤0.22 Gy and should be attributed to slight differences in the treatment plans. For the proximal airways maximum point dose is defined as 10 Gy/Fx and the volume exceeding 5 Gy/Fx should be <4 cm^3^. The evaluated proximal airways *D*_2_ ranged from 0.3 to 5.0 Gy for the ITV+5 conventional delivery. The potential of OAR dose decrease makes MLC tracking an interesting option, especially considering that SBRT might become an alternative for surgery^37^ in the near future. Decreasing toxicity of lung SBRT while maintaining the high control rate will make the therapy applicable to younger patients than currently exposed to the hypofractionated regiment.

Decreasing the PTV size while planning with a constant *D*_95_ prescription of 18 Gy/Fx results in a decreasing GTV dose. This is caused by the nonuniform dose profiles. It is unclear how these results influence local control. However, even when the MUs of the respective plans are scaled up to achieve GTV doses similar to the ones resulting from the RTOG 1021 prescription, we could show that OAR dose would still clearly decrease for most DVH points assessed in this study. Proper dose description for tracked treatments should include GTV constraints to benefit from the motion compensation.

For the patient data in this study, we could show that GTV *D*_98_ hardly changes for the midV plans when comparing the conventionally reconstructed dose to the static (planned) dose. We have also compared the midV plans to the ITV+5 reference. The dose difference for GTV Δ*D*_98_ comparing the conventionally delivered midV to ITV+5 was ≤0.42 Gy. The maximum dose deviation between the conventionally delivered midV and ITV+5 for spinal cord and proximal airways *D*_2_ was small: 0.25 Gy. A clear dose difference, however, was observed for lung *V*_20_: for the two patients with the largest tumor motion (patient 2 and 3), the midV plans show a decrease of 1.4%–1.5%.

Peulen *et al.* ^38^ have shown in a clinical study that the midV/midP approach in combination with online image‐guidance results in a high local control rate. High‐dose volumes were smaller for patients with substantial tumor motion and hence midV/midP must be considered an improvement over the conventional ITV approach. Our observation of the clear reduction in lung *V*_20_ for a patient with large tumor motion illustrates this finding. Not being limited to the motion parameters assessed by *a priori* 4D imaging, MLC tracking is expected to handle substantial baseline drifts better than midV/midP.

When aiming for decreasing the PTV, it should be noted that intrafractional motion is not the only reason for applying PTV margins. Setup errors are expected to be compensated by MLC tracking, but do influence the accuracy of online dose reconstruction. They should be minimized by in‐room imaging prior to treatment delivery. Delineation uncertainty is a larger problem as reducing margins is only acceptable when all tumor cells are encompassed by the GTV boundary. Optimal margin strategies for robust MLC tracking plans need to be further investigated, but are considered out of scope for this study.

### 4.C. Impact and future work

To our knowledge there is currently no other work published on 4D dose reconstruction incorporating intrafractional motion to assess tracked treatments. Work by other groups focuses on offline dose reconstruction for lung or abdominal sites. Glitzner *et al.* ^21^ also use DVFs to accumulate dose on a reference phase, however, they adopt DDM instead of EMT. In contrast to this group, our dose computation and accumulation algorithms run in ≤40 ms on average and hence are suitable for use in online dose reconstruction scenarios.

The MTV concept considers motion compensation of rigid shifts only and should be considered conservative for tumors with substantial deformation. For example, if the tumor expands in a single 4DCT phase only, this would propagate to an increased target volume for all phases. Future work should assess more sophisticated PTV definitions, robust to the actual patient motion.

It should be noted that this study is focused on the technical feasibility aspects while its potential clinical impact still needs to be analyzed in more detail. However, real‐time 4D dose reconstruction serves as a platform to further develop MLC tracking. Extending rigid tumor tracking to deformation tracking will potentially allow tracked treatment for more advanced lung tumors.

In this study, treatment planning was performed according to RTOG 1021 for an ITV/midV approach. Taking into account small local dose features during planning (like pushing spinal cord *D*_2_) makes sense to comply with RTOG, but not necessarily for a tracking scenario in which treatment fields are moved intentionally. The trade‐off between tight conformal target dose and optimal OAR sparing in terms of DVH points should be assessed.

Sonke *et al.* ^39^ have shown that although the shape of the tumor trajectory was found to be stable interfractionally, baseline shifts might invalidate the 4DCT planning dataset. Although MLC tracking is expected to cope with these shifts (depending on the motion acquisition method), this might result in a mismatch with the dose‐influence data. Therefore, to apply the proposed online 4D dose reconstruction method, the validity of the dose‐influence data should be assessed prior to each fraction. To allow for dose reconstruction on anatomy not captured on 4DCT, patient models could be utilized.^40^ Innovations in image‐guided radiation therapy technology like the MR‐linac^41^ may ultimately bring 4D online‐imaging and have the potential to continuously update a patient model, robust to baseline shifts and other anatomy changes. Extending this work to volumetric arc therapy (VMAT) is hindered by the substantial increase in dose‐influence data. We are currently investigating the trade‐off between the number of beam angles and the dosimetric accuracy for online dose reconstruction of VMAT deliveries. Generating dose‐influence data on the fly is currently too slow and we therefore envision future online 4D dose calculation directly, based on the patient model geometry and a real‐time Monte Carlo dose calculation.

## 5. CONCLUSIONS

We have implemented and evaluated a software platform for 4D online dose reconstruction. We have shown that dose can be calculated and accumulated in real‐time at 25 Hz for the whole lung volume using a clinical voxel resolution utilizing precalculated dose‐influenced data and DVFs. We could demonstrate for a limited patient cohort how decreased PTV margins lead to inadequate target coverage during untracked delivery for patients with substantial motion. Moreover, we observed that MLC centroid tracking successfully recovers the GTV target dose for these patients. OAR doses were consistently reduced by reducing PTV margins.

## ACKNOWLEDGMENTS

We acknowledge support from Elekta AB under a research agreement. Research at The Institute of Cancer Research is also supported by Cancer Research UK under Program No. C33589/A19727. MFF is supported by Cancer Research UK under Program No. C33589/A19908. We acknowledge NHS funding to the NIHR Biomedical Research Center at The Royal Marsden and The Institute of Cancer Research.

## CONFLICT OF INTEREST DISCLOSURE

The authors have no COI to report.
